# Initiated *Babesia ovata* Sexual Stages under In Vitro Conditions Were Recognized by Anti-CCp2 Antibodies, Showing Changes in the DNA Content by Imaging Flow Cytometry

**DOI:** 10.3390/pathogens8030104

**Published:** 2019-07-17

**Authors:** Thu-Thuy Nguyen, Minh-Anh Dang-Trinh, Luna Higuchi, Juan Mosqueda, Hassan Hakimi, Masahito Asada, Junya Yamagishi, Rika Umemiya-Shirafuji, Shin-ichiro Kawazu

**Affiliations:** 1 National Research Center for Protozoan Diseases, Obihiro University of Agriculture and Veterinary Medicine, Inadacho, Nishi 2-13, Obihiro, Hokkaido 080-8555, Japan; 2 Research Center for Global Agromedicine, Obihiro University of Agriculture and Veterinary Medicine, Inadacho, Nishi 2-13, Obihiro, Hokkaido 080-8555, Japan; 3 Natural Sciences College, Autonomous University of Querétaro, 76230 Queretaro, Mexico; 4 Department of Protozoology, Institute of Tropical Medicine, Nagasaki University, 1-12-4 Sakamoto, Nagasaki 852-8523, Japan; 5 Research Center for Zoonosis Control, Hokkaido University, North 20, West 10 Kita-ku, Sapporo, Hokkaido 001-0020, Japan

**Keywords:** *Babesia ovata*, DTT, imaging flow cytometry, sexual stage induction

## Abstract

Sexual stage induction under in vitro conditions is useful for biological and molecular studies of *Babesia* parasites. Therefore, in the present study, we induced *B. ovata* tick stages using the chemical inducers: xanthurenic acid (XA), dithiothreitol (DTT) and tris (2-carboxyethyl) phosphine (TCEP) at 27 °C or 37 °C conditions. Cultures at low temperature (27 °C) or treated with XA/TCEP induced a large number of extra-erythrocytic merozoites, which transformed into round shape cells at 12–24 h post-induction (pi). However, typical forms of tick stages (aggregation forms and the spiky forms/ray bodies) were only observed in the cultures treated with 40 mM or 60 mM of DTT during 3–6 h pi. The induced cells were recognized by anti-CCp2 rabbit antisera. DNA content of the cell population treated with 40 mM of DTT was analyzed by imaging flow cytometry at 0, 12 and 48 h pi. The results indicated that the parasite population with diploid-like double DNA content increased at 48 h pi. Our observations on morphological and changes in the DNA content provide useful information for understanding the life cycle of *B. ovata* under in vitro conditions, which will facilitate further studies on basic biology and the development of transmission blocking vaccines against bovine babesiosis.

## 1. Introduction

Babesiosis is a tick-transmitted disease of animals manifested by anemia and occasional hemoglobinuria caused by the protozoan parasites of the genus *Babesia* [1]. Several *Babesia* spp. are known to infect cattle, with an estimation of 1–2 billion cattle worldwide currently exposed to one or more babesiosis pathogens [2,3]. Bovine babesiosis has considerable economic impact related to losses in milk and meat production, abortions, and a general impact on the global cattle trade industry [4]. *Babesia bigemina* and *B. bovis* are the most important species in terms of economic and veterinary significance. *B. ovata* is among the low pathogenic species, however, infection may lead to severe conditions in cattle when co-infected with *Theileria orientalis* [5]. *B. ovata* infection is endemic in Japan, South Korea, China, Mongolia and Thailand. The disease is transmitted by the tick vector *Haemaphysalis longicornis* which is widely distributed in Asia Pacific regions [6].

The lifecycle of *Babesia* spp. is complex and consists of four main processes which results in the parasite’s persistence in the environment, namely “schizogony” and “merogony” are when the parasites multiply asexually in the vertebrate host’s erythrocytes, followed by “gamogony” and “sporogony” which refer to parasite sexual and asexual reproduction in the gut and salivary gland of the tick vector, respectively. Although the basic lifecycle is understood, limited knowledge on tick-stage development of *Babesia* spp. has been elucidated to date. Further understanding on the biology of transmission, parasite development and persistence in the tick, as well as parasite–tick interactions are important to design the future efforts of disease control focusing on tick-stage parasites such as transmission-blocking vaccines.

Such kinds of “parasite–tick” research is usually restricted by the absence of effective laboratory transmission models. To overcome this issue, sexual stage induction under in vitro conditions has been proven to be useful and feasible. Gough et al. [7] were the first research group to initiate sexual development of *B. bigemina* in vitro, using gut homogenate from female ticks, *Boophilus micropliis*. Thereafter, Mosqueda et al. [8] reported utilization of a chemical inducer and low temperature. The established method was modified and applied extensively in a number of studies, such as, the study of Hussein et al. [9] which characterized the *hap*2 gene expression and function during sexual development of *B. bovis*. Recently, the *ccp* gene family and a putative *methyltransferase* gene of *B. bigemia* were identified as novel biomarkers for parasite tick stages [10]. In *B. ovata*, in vitro sexual stage development was performed; however, it required tick midgut contents [11]. A simpler alternative method is still needed. Therefore, in the present study, we induced *B. ovata* sexual stages using chemical inducers and induction temperatures: xanthurenic acid (XA), dithiothreitol (DTT) and tris (2-carboxyethyl) phosphine (TCEP) at 27 °C and 37 °C. In addition, the stage-specific expression of CCp2 which was confirmed as sexual-stage-specific molecular marker in *B. bigemina* and *B. divergence* [12,13] was examined in our in vitro induced cells. The morphological and changes in the DNA content, together with the CCp2 expression in the induced tick stages, are described for the first time.

## 2. Results

### 2.1. Development of B. ovata Tick Stages under In Vitro Induction

**Figure 1:**
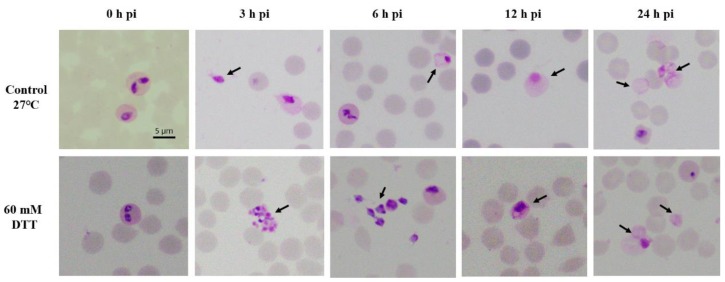
Development of *B. ovata* tick stages at different incubation time post-induction (pi). Control group (without addition of chemical inducers) and dithiothreitol (DTT) group were cultured at 27 °C. Free merozoites and tick stages are indicated with arrows. Scale bar: 5 µm.

*B. ovata* cultures were exposed to either XA, DTT or TCEP and incubated at two different temperatures: 37 °C in the presence of 5% CO_2_ or 27 °C in air (Supplementary Table S1). As controls, the parasites were cultured with only fresh medium and incubated at the same temperature conditions. The control group of *B. ovata* at 37 °C did not show any morphological changes; however, transformation of *B. ovata* from intraerythrocytic parasites to extracellular forms was observed in all of other groups (control group at 27 °C and groups with addition of chemical inducers). Low temperature (27 °C) initiated a large number of emerging merozoites at 3 h post-induction (pi), then transformed into large round-shape forms during 6–24 h pi (Figure 1 top panels). XA and TCEP induced the morphological changes of the parasites similar to that observed in the control at 27 °C (data not shown). In addition, the numbers of different extracellular forms observed at 27 °C control, XA and TCEP groups were not statistically different (*p*-value > 0.05). DDT was the only chemical that could induce a variety of typical forms of sexual-stage, including aggregation forms and ray bodies (Figure 1 bottom panels, Supplementary Table S1). With an addition of 40 or 60 mM of DTT in the culture, *B. ovata* developed into different extracellular forms (described below):

From 0–3 h pi: A high number of free merozoites were found. The results showed that the highest percentage of extracellular merozoites (1.4%) was seen at this time point (Table 1). Aggregation forms were observed and consisted of extracellular spherical forms in a round shape with a size of 1.5–2.0 µm (Figure 1 bottom panels). Aggregation forms were then hardly seen beyond 3 h pi in the induced culture (Table 1).

At 6 h pi: The spiky forms/ray bodies appeared, having one or two nuclei, and several short projections of 3–4 µm in size (Figure 1 bottom panels). Free merozoites remained in high number in the culture.

At 12 h pi: The cells transformed into large, round forms (5–8 µm) with one or two nuclei and clear cytoplasm (Figure 1 bottom panels), as seen in the control group at 12–24 h pi (Figure 1 top panels). Their nuclei were located in the peripheral body. 

At 24 h pi, the nuclei of the round shape forms were hardly seen under light microscopy, revealing cells in large round shape (4–8 µm) and clear cytoplasm (Figure 1 top and bottom panels). No further development of cells was observed beyond 24 h pi. The transformation of *B. ovata* from merozoites to tick-stage forms, either induced by low temperature, XA, TCEP or DTT, was asynchronous. Although induced cultures were a mixture of several morphological forms, large and round parasites became dominant with percentage values of 4% at 48 h pi (Table 1).

**Figure 2:**
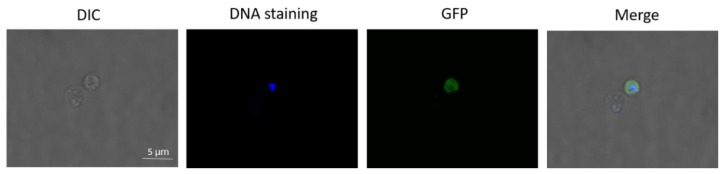
Live fluorescence microscopy of sexual stages of GFP-expressing *B. ovata*. Sexual stages at 48 h post-induction were purified by Percoll gradient, nucleus stained with Hoechst and visualized by confocal microscope. Scale bar: 5 µm. DIC: differential interference contrast; DNA staining: DNA staining with Hoeschst 33342; GFP: green fluorescent protein; Merge: merged image of DNA staining, GFP and DIC.

The *B. ovata* tick stages induced in vitro were enriched by Percoll gradient. To confirm viability of the cells, green fluorescent protein (GFP)-expressing line at 48 h pi (mostly round form with one or two nuclei) were incubated with Hoechst 33342 for DNA staining and observed under confocal microscope. The cells expressed green fluorescence in their cytosol consistently, which confirmed that “the sexual stages” we observed were alive with intact cell membrane but not dead and degraded cells (Figure 2).

### 2.2. B. ovata Tick Stages Induced under In Vitro Conditions Were Recognized by Sexual-Stage-Specific Anti-Ccp2 Antibody 

**Figure 3:**
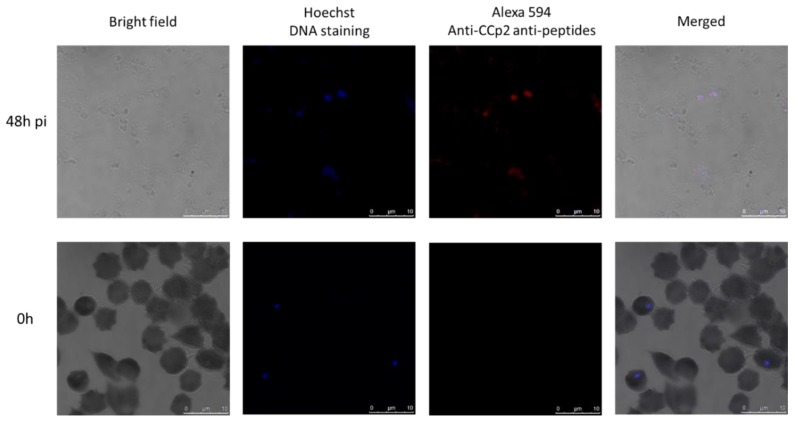
Indirect immunofluorescence antibody test (IFAT) demonstrating expression of sexual-stage-specific CCp2 protein in in vitro induced *B. ovata* extracellular cells at 48 h pi. The intraerythrocytic merozoites did not show any immunoreactivity with anti-CCp2 antisera.

To verify the in vitro induced *B. ovata* tick stages, we selected CCp2 as a sexual-stage-specific marker. Samples from 0 h and 48 h pi were immunostained with antisera against CCp2 peptides. The results showed that extracellular cells induced in vitro (sample at 48 h pi) were recognized by the antisera; while intraerythrocytic merozoites (sample at 0 h) did not show any immunoreactivity (Figure 3).

### 2.3. Measuring DNA Content in B. ovata Tick Stages

**Figure 4:**
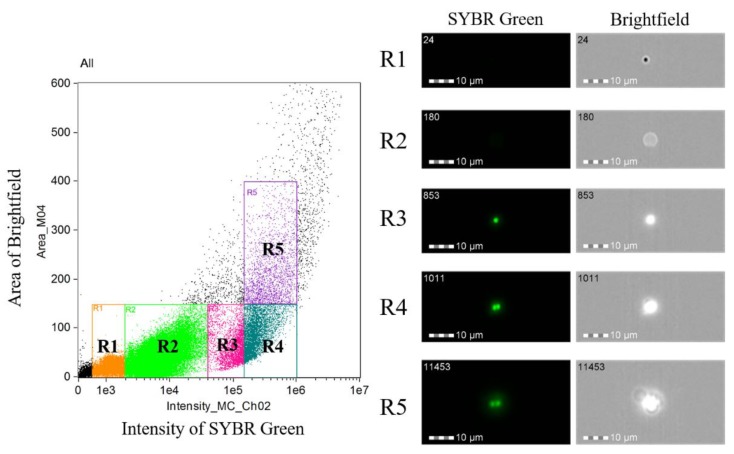
Imaging flow cytometry analysis of *B. ovata* before sexual-stage induction. Parasite DNA was stained with SYBR green I. Five populations were detected on the basis of SYBR green intensity: R1: speed beads; R2: non-infected red blood cells (RBCs); R3: merozoites with a single fluorescent dot (1n); R4 and R5: budding and binary forms showed diploid-like double DNA content (2n).

**Figure 5:**
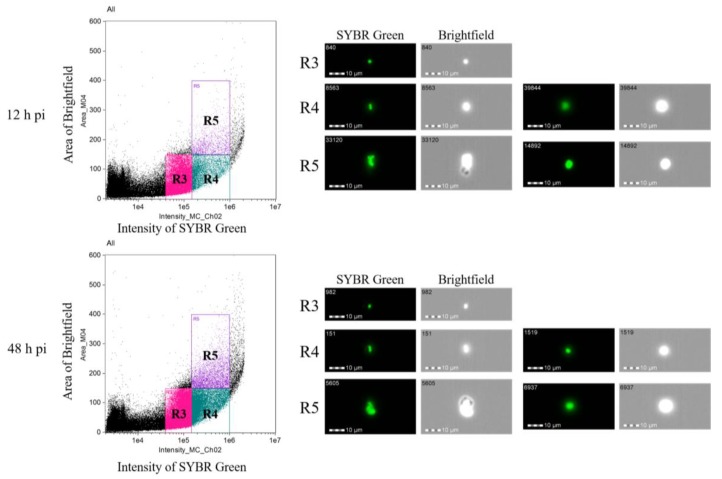
Imaging flow cytometry analysis of *B. ovata* sexual stages at 12 h and 48 h post-induction. The haploid population R3 were free merozoites and gametocytes. The populations with diploid-like double DNA content were R4 and R5. R4 consisted of two cell types: cells with two nuclei were supposed to be from two gametes before cell fusion; and cell with one nucleus was supposed to be zygote. R5 mostly consisted of aggregation forms.

To determine changes in the DNA content before and after the tick-stage induction, DNA content of the parasite cell was monitored with imaging flow cytometry analysis (Figure 4). Initially, we analyzed the 0 h pi sample to determine the parasite populations. As shown in Figure 4 and Figure 5, populations (R1–R5) were determined in the sample. The R1 population represented speed beads used for imaging flow cytometry, and R2 population represented uninfected erythrocytes. R1 and R2 population were negative for SYBR green fluorescence. Contrary, clear SYBR green fluorescence was detected in R3–R5 populations. The single fluorescent dots in R3 population indicated that this population represented the infected erythrocyte with single merozoite, and double fluorescent dots in R4 population represented an infected erythrocyte with binary form. The R5 population consisted of cells with a higher bright-field in which agglutinated erythrocytes with parasite were observed. We adopted this gating as the criteria for analyzing the tick-stage induced samples with 40 mM of DTT. The 12 h pi sample showed 16,680, 4709 and 1174 cells in R3, R4 and R5 populations, respectively (Table 2). The image of R3 population showed single SYBR green fluorescent dots as expected, and these cells might represent free merozoites and ray bodies (Figure 5). R4 population showed a mixture of cells with single and double fluorescence dots and relatively larger cytoplasm than R3 and could represent big round cells. These big round cells were seen in R5 population together with many agglutinated cells that might represent aggregation forms. The similar images were obtained in R3–R5 populations in 48 h pi sample. The number of the cells in 48 h pi sample was 27,428, 14,603 and 3447 for R3, R4 and R5 populations, respectively. The ratio of the cells with higher DNA contents (R4, R5) was increased in 48 h pi samples as compared with 12 h pi samples which was in good accordance with the findings obtained under the microscopic observations with Giemsa-stained samples (Table 1). Meanwhile, 60.3% of cells were still in the R3 fraction and this rate was higher than that observed in the Giemsa-stained samples (Table 1). The enrichment step of extra-erythrocyte parasites might contribute to the difference of these results.

## 3. Discussion

Sexual stage induction under in vitro conditions was applied in a number of studies to identify and evaluate the sexual-stage-specific molecules with potential as a transmission-blocking vaccine to *B. bigemina* and *B. bovis* [9,10]. It also serves as a complimentary method to elucidate some unknown aspects on the complex life cycle of the parasites during sexual stage development. Although initial development of *B. ovata* in the tick midgut was described previously by Higuchi et al. [14] and Maeda et al. [11], our study succeeded to establish *B. ovata* tick-stage induction merely under in vitro conditions. The initiated *B. ovata* tick stages were recognized by rabbit antisera against CCp2. This reactivity is consistent with the probability that those cells have undergone sexual differentiation, based upon the expression patterns of this protein in the tick stage of *B. bigemina* and *B. divergence* [12,13]. In addition, they considerably showed compatible morphological changes with DNA content.

Higuchi et al. [14] described the presence of “ring forms” (2–3 µm), then spherical forms (4–5 µm) inside the midgut of *H. longicornis* right after erythrocyte degradation. We also found spherical forms (with round or leaf shape, 4–5 µm) in mixture with a large number of free merozoites in the in vitro culture as early as 3–6 h pi (Supplementary Figure S1a) and counted them as free merozoites in this study. The *B. ovata* aggregation forms and ray bodies were not described in tick midgut [14]; however, they were reported in vitro [11]. Our observation is similar with these previous reports in terms of morphology, timing of their appearance, and that aggregation forms were necessary for the development of ray bodies in *B. ovata*. The large round shape forms developed from ray bodies at 12–24 h pi in this study are considered zygotes, since Higuchi et al. also described zygotes in tick midgut as large round or elliptic form, with the nucleus located in the peripheral of the body and light cytoplasm staining [14]. However, the forms “considered zygotes” that were seen in our in vitro culture were smaller (5–8 µm) than in the midgut (9–10 µm). No further transformation was seen after “zygotes” formation, although we also observed a cell form (in very low frequency), with long projection, and clear cytoplasm similar to the mature ray body reported in the study of Maeda et al. (Supplementary Figure S1b). Additionally, quantification of the DNA content using an imaging flow cytometer supported our observations. The initial *B. ovata* culture was found to contain two populations: one with haploid (which were merozoites) and another with diploid-like double DNA content (were supposed to be budding forms or trophozoites). At 12 h pi, the haploid cells population was 73.9% of the parasites which were free merozoites and the form “considered gametocytes”. On the other hand, at 48 h pi sample, ratio of the cells with diploid-like double DNA content was increased, and this population contains “round zygotes”. According to microscopic examination, aggregation forms were hardly seen after 3 h pi, only once in a single sample at 48 h pi (Table 1); while imaging flow cytometry revealed 5.2% and 7.6% of aggregation forms in 24 h and 48 h pi, respectively (Table 2 and Figure 5). This can be explained by the asynchronous state of in vitro sexual stage induction; therefore, aggregation forms might appear later at 24 h or 48 h, but in very low numbers that hardly could be seen by light microscopy. On the other hand, in flow cytometry, the samples were purified to obtain only extracellular cells for analysis, resulting in a higher percentage of the aggregation forms than its actual numbers in blood smear observation. Further analysis of the population with higher DNA content is required since it also contained other types of cells such as fusing cells and aggregation forms. DNA measurement was employed to confirm the developmental stages within the lifecycle of different *Babesia* spp. [15,16]. Among various available methods of DNA measurement, our study demonstrated that imaging flow cytometry is useful and accurate for phenotyping cell populations based on their DNA content, cell size and morphology.

Several factors were reported to induce in vitro development of tick stages in *Babesia* spp. They include temperature, CO_2_ concentration, pH, XA, DTT and TCEP; but the effects of these inducers are not the same in different parasite species [8,10]. The results of our study showed that in *B. ovata*, low temperature (27 °C) or addition of XA/TCEP triggered transformation of intraerythrocytic parasites to extracellular round shape cells. However, only in the presence of 40 mM or 60 mM of DTT, aggregation forms and ray bodies (Strahlenkörper) with short projections were observed distinctively. Since zygotes were observed, there could be other tick stages in (27 °C) and/or XA/TCEP conditions. However, these conditions might have induced only few numbers of aggregation forms and ray bodies in the culture or their morphology was atypical such that light microscopy did not allow their reliable recognition [17]. On the other hand, DTT might induce a higher number of these forms that could be detected easily.

We observed no further developmental changes after “zygote” appearance in the in vitro culture, while tick midgut contents could stimulate sexual transformation up to vermicular forms [11]. Another constraint of in vitro induction would be the asynchronous transformation. Therefore, it is difficult to obtain a single specific sexual form in the culture at 0–24 h pi, except after 48 h when *B. ovata* zygotes become dominant. Apart from these issues, we found in vitro induction of *B. ovata* tick stages a simple and quick method and could facilitate further studies on elucidating molecular development of tick stages of this *Babesia* species.

## 4. Materials and Methods

### 4.1. In Vitro Induction of B. ovata Tick Stages

*B. ovata* (Miyake strain) wild type and green fluorescent protein (GFP)-expressing parasite line (D11R) [18] were maintained in vitro with purified bovine red blood cells (RBC, Nippon Bio-Supply Center, Tokyo, Japan) and GIT medium (Nihon Pharmaceutical Co., Tokyo, Japan) supplemented with 40% fetal bovine serum (Biowest, MO, USA) using a microaerophilic stationary-phase culture system [19]. Tick stages were induced by adding either xanthurenic acid (XA) (Wako, Osaka, Japan), dithiothreitol (DTT) (Wako, Osaka, Japan) or tris (2-carboxyethyl) phosphine (TCEP) (Sigma-Aldrich, MO, USA) at serial concentrations: 0, 25, 50, 100, 125, 250, 500 and 1000 µM (XA), and 0, 20, 40, 60, 80, 100 mM (DTT/TCEP). The induced in vitro cultures were then incubated at 27 °C or 37 °C, with or without CO_2_ according to the protocols of Mosqueda et al. [8] and Bohaliga et al. [10] (Supplementary Table S1). A control group using only fresh medium was maintained at the same temperature conditions. Three independent experiments were carried out. One sample from each culture group was taken every 3 h from 0 h up to 24 h, then 48 h and 72 h post-induction (pi) and used for Giemsa-stained smear analysis. Parasitemia and different cell forms were quantified by counting the parasites/cells in 1000 erythrocytes three times. Data were statistically analyzed using GraphPad Prism 6. Fisher’s exact test was used to compare parasitemia and rate of different sexual cell forms. The difference was considered to be statistically significant if *p*-value < 0.05. In vitro cultures of *B. ovata* D11R were examined under fluorescent microscopy to confirm expression of GFP and verify cell viability.

### 4.2. Synthetic Peptides and Antisera

Protein sequence of *B. ovata* CCp2 was retrieved from EupathDB (https://piroplasmadb.org/piro/), under Gene ID: BOVATA_030410 [20]. Synthetic peptides were designed and generated as: CCp2.1: GEHDKFNEAPVGRVVKASC (aa 785–803) and CCp2.2: DGSIEPSMALLKGGRSC (aa 1302–1318). Rabbit antisera were obtained against CCp2 peptides (Sigma Aldrich, Tokyo, Japan). The antisera titers were assessed by indirect enzyme-linked immunosorbent assay (ELISA) with the immunized peptides as antigens and stored in −20 °C until use. 

### 4.3. Immunofluorescence Assays

Immunofluorescence assays were performed using *B. ovata* sexual stage induced culture at 0 h and 48 h pi. The induced sexual staged in the sample at 48 h. The samples were washed 3 times with cold phosphate buffed saline (PBS) before applying as thin smears on the glass slides. The slides were air dried for 30–45 min, fixed in methanol at −20 °C for 30 min, then blocked in blocking buffer (PBS containing 10% of normal goat serum) at 37 °C for 30 min in a humid chamber. After washing 3 times with PBS, the slides were incubated with anti-CCp2 rabbit antisera (CCp 2.1 and CCp 2.2) (1:20, diluted in blocking buffer) at 37 °C for 30 min. The slides were immunostained with Alexa Fluor 594 conjugated goat-anti-rabbit IgG secondary antibody (1:1000) (Thermo Fisher Scientific, Waltham, MA, USA) and incubated at 37 °C for 30 min. DNA was stained with Hoeschst 33342 (1:1000) (Dojindo, Kumamoto, Japan) for 5 min at 37 °C, and the slides were observed under a confocal microscope (Leica TCS SP5, Leica Microsystems, Wetzlar, Germany).

### 4.4. Imaging Flow Cytometry Analysis of B. ovata Tick Stages

For imaging flow cytometry analysis, *B. ovata* in vitro cultures were treated with 40 mM of DTT and sampled at 0, 12 and 48 h pi. To enrich and purify the extra-erythrocyte parasites, Percoll gradient centrifugation was performed for 12 and 48 h pi samples. Briefly, 2 mL of in vitro culture was layered on top of 10 mL of 47% Percoll and centrifuged at 12,000 *g* for 20 min at 4 °C. The medium-Percoll interphase was collected, washed twice with 5 times volume of cold PBS, and suspended in 500 µL of cold PBS. The parasites’ DNA was stained with SYBR green I (Lonza, ME, USA) at 1:500 dilutions for 20 min on ice, then washed twice with PBS. The DNA stained samples were subjected to ImageStream X Mark II (Merck Millipore, Burlington, MA, USA) with the default setting. One hundred thousand data was obtained from 0 h pi sample and speed beads, uninfected erythrocytes, and each parasite population were determined. Since speed beads showed <2000 SYBR green fluorescence intensity, data of 100,000 cells with ≥2000 SYBR green fluorescence intensity were obtained for 12 and 48 h pi samples. The obtained cytometric data were analyzed by IDEAS 6.2 software.

## Figures and Tables

**Table 1:** Parasitemia and proportion of different sexual stages of *B. ovata* in vitro culture at different time points post-induction in the presence of 60 mM DTT at 27 °C.

*B. ovata*	0 h	3 h	6 h	9 h	12 h	24 h	48 h	72 h
Parasitemia (%)	4.50 ± 0.40	3.50 ± 0.00	3.00 ± 0.00	2.10 ± 0.10	1.90 ± 0.15	1.20 ± 0.10	0.50 ± 0.00	0.40 ± 0.20
Free merozoites (%)	0.19 ± 0.01	**1.40** ± 0.00 *	**1.40** **± 0.00**	0.35 ± 0.03	0.40 ± 0.00	0.25 ± 0.03	0.10 ± 0.00	-
Aggregation forms (%)	-	**0.07** **± 0.00**	-	-	-	-	0.03 ± 0.00	-
Gametocytes ** (%)	-	-	0.20 ± 0.00	0.10 ± 0.00	0.30 ± 0.10	**0.6** **± 0.1**	0.10 ± 0.00	-
Zygotes *** (%)	-	-	-	0.18 ± 0.00	0.4 ± 0.1	0.75 ± 0.2	**4.00** **± 0.10**	2.00 ± 0.00

The percentage was calculated by counting the number of merozoites/sexual forms in 3000 erythrocytes in three independent experiments. * Bold indicates the highest percentage of each sexual form. ** Cells with one nucleus or two nuclei and short projections are considered gametocytes/ray bodies. *** Round, big cells with clear cytoplasm are considered zygotes.

**Table 2:** Number of cells in each population analyzed by imaging flow cytometer.

	12 h pi	48 h pi
R3	16,680 (73.9%)	27,428 (60.3%)
R4	4709 (20.9%)	14,603 (32.1%)
R5	1174 (5.2%)	3447 (7.6%)

## Supplementary Materials

The following are available online at https://www.mdpi.com/2076-0817/8/3/104/s1, Table S1. Morphological changes of *B. ovata* sexual stages observed in different induction conditions. Table S2. Parasitemia and proportion of different sexual stages of *B. ovata* in vitro culture at different time point post induction at 27 ℃. Figure S1. Extracellular cell forms in 60 mM DTT induced culture (a) Spherical forms at 3h pi. (b) Cell with long projection and clear cytoplasm might be a mature ray body 24h pi.
