# Supplementary material for: Initiated Babesia ovata Sexual Stages under In Vitro Conditions Were Recognized by Anti-CCp2 Antibodies, Showing Changes in the DNA Content by Imaging Flow Cytometry
Source: Pathogens. 2019 Jul 17;8(3):104. doi: 10.3390/pathogens8030104 (PMC6789521; doi:10.3390/pathogens8030104)
Supplement: Supplementary file 1 [file pathogens-08-00104-s001.pdf]

Supplementary Materials:

Table S1. Morphological changes of *B. ovata* sexual stages observed in different induction conditions.

Inducer	Incubation temperature	
	37 ℃ with 5% CO_2	27 ℃ in air
XA	Gametes and zygotes	Gametes and zygotes
TCEP	Gametes and zygotes	Gametes and zygotes
DTT	Aggregation forms, ray bodies, gametes and zygotes	Aggregation forms, ray bodies, gametes and zygotes
Control	None	Gametes and zygotes

Abbreviations: XA, Xanthurenic acid; TCEP, Tris(2-carboxyethyl)phosphine; DTT, Dithiothreitol.

Table S2. Parasitemia and proportion of different sexual stages of *B. ovata in vitro* culture at different time point post induction at 27 ℃.

B. ovata	**0h**	**3h**	**6h**	**9h**	**12h**	**24h**	**48h**	**72h**
Parasitemia (%)	4.10 ± 0.60	2.9 ± 0.10	2.70 ± 0.50	2.50 ± 0.00	1.60 ± 0.20	1.20 ± 0.10	0.80 ± 0.20	0.30 ± 0.10
Free merozoites (%)	0.17 ± 0.02	**0.50 ± 0.05***	0.30 ± 0.07	0.20 ± 0.09	0.20 ± 0.03	0.25 ± 0.03	0.20 ± 0.10	-
Aggregation forms (%)	-	-	-	-	-	-	0.03 ± 0.00	-
Gametocytes** (%)	-	-	-	-	-	-	0.10 ± 0.00	-
Zygotes** *(%)	-	-	-	-	0.70 ± 0.07	1.80 ± 0.50	**2.4 ± 0.1**	-

The percentage was calculated by counting the number of merozoites/sexual forms in 3,000 erythrocytes in three independent experiments. *Bold indicates the highest percentage of each sexual forms. **Cells with one or two nucleus and short projections are considered gametocytes/ray bodies. ***Round, big cells with clear cytoplasm are considered Zygotes.

Nguyen et al., Supplementary Fig.1

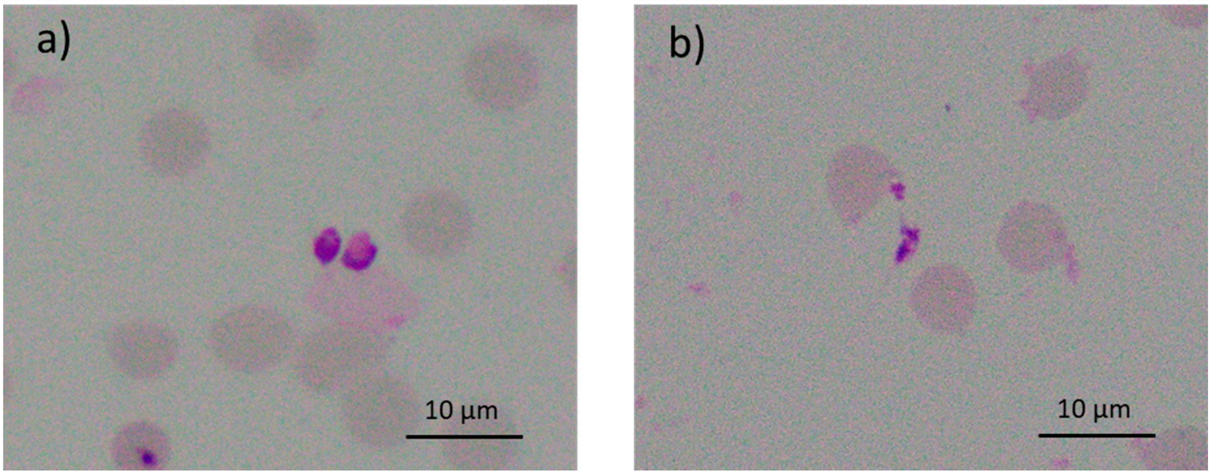

Figure S1. Extracellular cell forms in 60 mM DTT induced culture (a) Spherical forms at 3h pi. (b) Cell with long projection and clear cytoplasm might be a mature ray body 24h pi. .
